# Potent antibody immunity to SARS‐CoV‐2 variants elicited by a third dose of inactivated vaccine

**DOI:** 10.1002/ctm2.732

**Published:** 2022-02-27

**Authors:** Bin Ju, Bing Zhou, Shuo Song, Qing Fan, Xiangyang Ge, Haiyan Wang, Lin Cheng, Huimin Guo, Dan Shu, Lei Liu, Zheng Zhang

**Affiliations:** ^1^ Institute for Hepatology National Clinical Research Center for Infectious Disease Shenzhen Third People's Hospital Shenzhen China; ^2^ The Second Affiliated Hospital School of Medicine Southern University of Science and Technology Shenzhen China; ^3^ Guangdong Key Laboratory for Anti‐infection Drug Quality Evaluation Shenzhen China; ^4^ Department for Infectious Diseases National Clinical Research Center for Infectious Disease Shenzhen Third People's Hospital Shenzhen China; ^5^ Shenzhen Research Center for Communicable Disease Diagnosis and Treatment of Chinese Academy of Medical Science Shenzhen China

Dear Editor,

This study found that a third dose of inactivated vaccine largely increased plasma neutralization against SARS‐CoV‐2 variants including beta, delta, and lambda. More importantly, the high‐affinity anti‐RBD (receptor‐binding domain) memory B cells were also generated by the third vaccination, suggesting a more potent and longer protection.

The coronavirus disease 2019 (COVID‐19) pandemic has already lasted for nearly 2 years and continues to threaten human health and life. While several effective vaccines had been deployed to combat wild‐type (WT) virus infection,^1^, ^2^, ^3^ emerging SARS‐CoV‐2 variants with enhanced transmissibility significantly escaped the neutralization of vaccine‐elicited plasma. Breakthrough infections of variants after vaccination have occurred widely with a significant reduction in vaccine efficacy over time.^4^, ^5^, ^6^ Currently, many researchers have asked whether a third vaccination is necessary to increase the titres of neutralizing antibodies (nAbs) and to better control the COVID‐19 pandemic.^7^, ^8^ Here, we evaluated the antibody immunity to SARS‐CoV‐2 variants elicited by a third dose of inactivated vaccine in a cohort of volunteers.

In this study, 533 participants who received two or three doses of BBIBP‐CorV were enrolled and followed up at five follow‐up time points (Figure 1A). The mean value of plasma IgG to SARS‐CoV‐2 WT RBD was significantly increased 10.41‐fold at week 2 after the second vaccination. However, IgG values were gradually decreased to 41.8% at month 2 after the second vaccination and additionally dropped to 42.9% at month 7. Subsequently, we collected blood samples from a total of 176 individuals who accepted a third dose of BBIBP‐CorV. Their mean IgG values were increased eightfold compared with those before the third vaccination and 1.44‐fold compared with those after the second vaccination (Figure 1B). RBD‐specific IgM displayed a similar pattern of kinetics as IgG. However, the difference between IgM and IgG was that the third vaccination did not induce a strong IgM response (Figure 1C), suggesting that IgG may play a more important role in recalling to the SARS‐CoV‐2 vaccine. The results of 113 serially paired samples also confirmed the above dynamics of RBD‐specific plasma IgG and IgM (Figure 1D,E).

**FIGURE 1:**
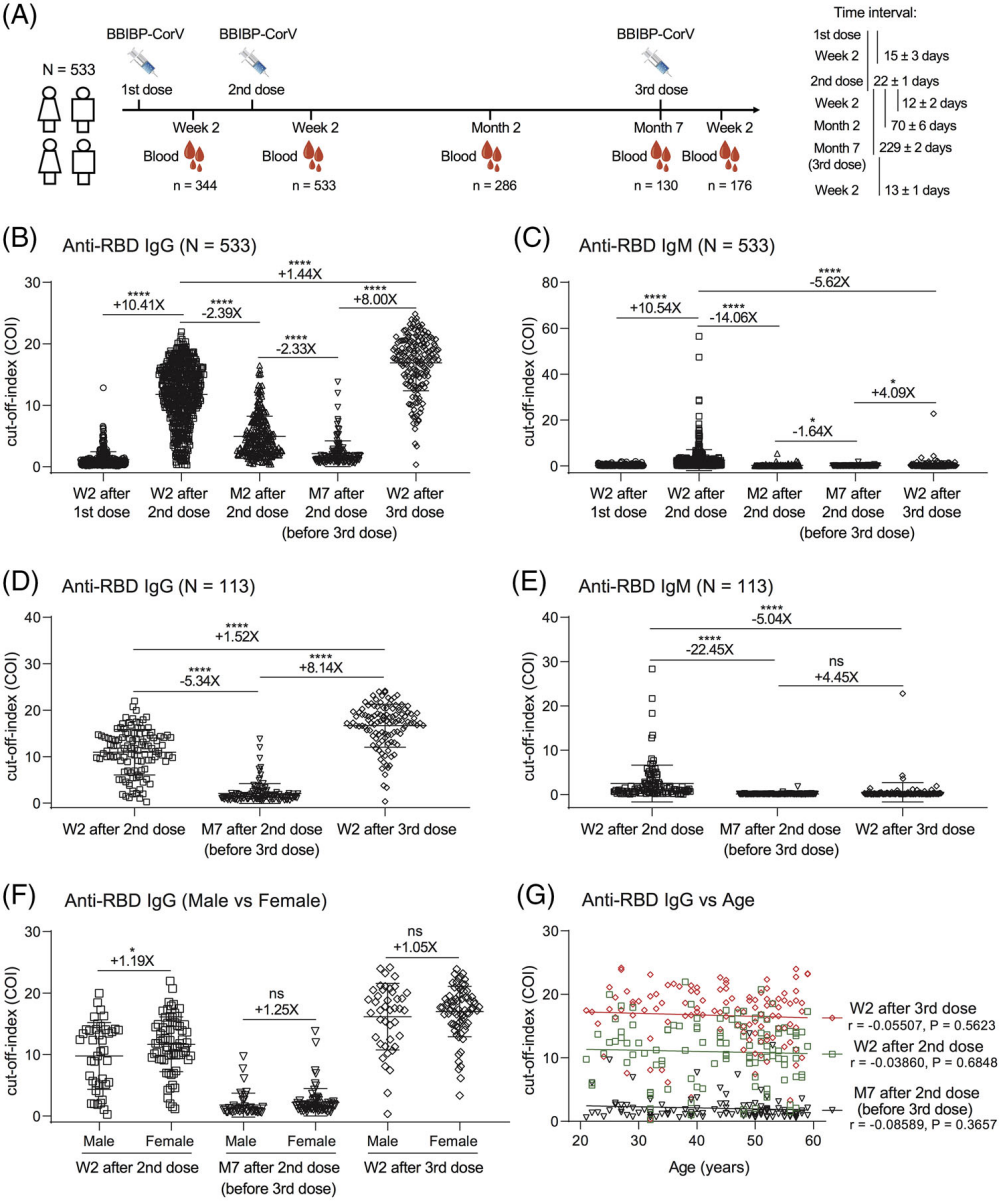
Longitudinal dynamics of humoral antibodies and boosting effect of the third dose of inactivated vaccine against SARS‐CoV‐2. (A) Immunization schedule and blood specimen collection of 533 donors who received two or three doses of inactivated vaccines in this project. The interval time is shown as the mean ± SD days. (B–C) Plasma antibody dynamics of anti‐RBD IgG (B) and IgM (C) during three doses of vaccines at multiple time points (*n* = 344, 533, 286, 130 and 176, respectively). (D–E) The binding ability of IgG (D) and IgM (E) to RBD from 113 donors who completed three time‐point follow‐up visits: week 2 and month 7 after second vaccination and week 2 after third vaccination (*n* = 113 in each time point). (F) Comparison of anti‐RBD IgG values between male (*n* = 42) and female (*n* = 71) vaccines. (G) Correlation analysis between anti‐RBD IgG values and ages in the 113 donors at three follow‐up visits. The correlation was analysed using GraphPad Prism 8.0 software by linear regression model. W2: Week 2, M2: Month 2, M7: Month 7, ‘+’ indicates increased antibody value, ‘–’ indicates decreased antibody value. ‘X’ indicates fold change. *p < .05; ****p < .0001; ns, not significant

We further compared the differences in anti‐RBD IgG levels between male and female donors. As shown in Figure 1F, male and female donors displayed similar levels of RBD‐specific IgG after the third vaccination, although female donors had higher levels of IgG than males at week 2 after the second vaccination. Meanwhile, there were no obvious relationships between IgG values and ages at three different follow‐up time points (Figure 1G). These data showed that the third dose of inactivated vaccine induced robust binding antibodies to SARS‐CoV‐2 independent of gender and age.

To evaluate the ability of a third vaccination to fight against the infection of mutant viruses, we established the enzyme‐linked immunosorbent assay and neutralization assay to detect binding antibodies and nAbs against WT, beta, delta, and lambda variants (Figure S1). Similar to the binding response to WT RBD, mutated RBD‐specific IgG was sharply decreased at month 7 after the second vaccination but was significantly increased by the third vaccination (Figure 2A). The plasma neutralizing activities displayed the same patterns as their binding activities (Figure 2B). At week 2 after the second vaccination, over 95% of plasma demonstrated effective neutralization against WT virus and also, to some extent, maintained neutralizing activities against three important variants. However, at month 7 after the second vaccination, nearly half of the plasma (49/113) lost their neutralizing activities, and the mean inhibition was decreased to 53.6% against the WT strain. Notably, the inhibitions of plasma against beta, delta and lambda variants had decreased to 34.4%, 40.4% and 44.8%, indicating their poor defences. After the third vaccination, the plasma inhibitions were significantly increased to 94.6%, 71.6%, 83.4% and 89.0% within 2 weeks, respectively. In addition, there were strong correlations between plasma inhibitions and their binding activities (Figure 2C; Figures S2 and S3). These findings indicated that the third dose of inactivated vaccine‐elicited potent nAbs against SARS‐CoV‐2 variants.

**FIGURE 2:**
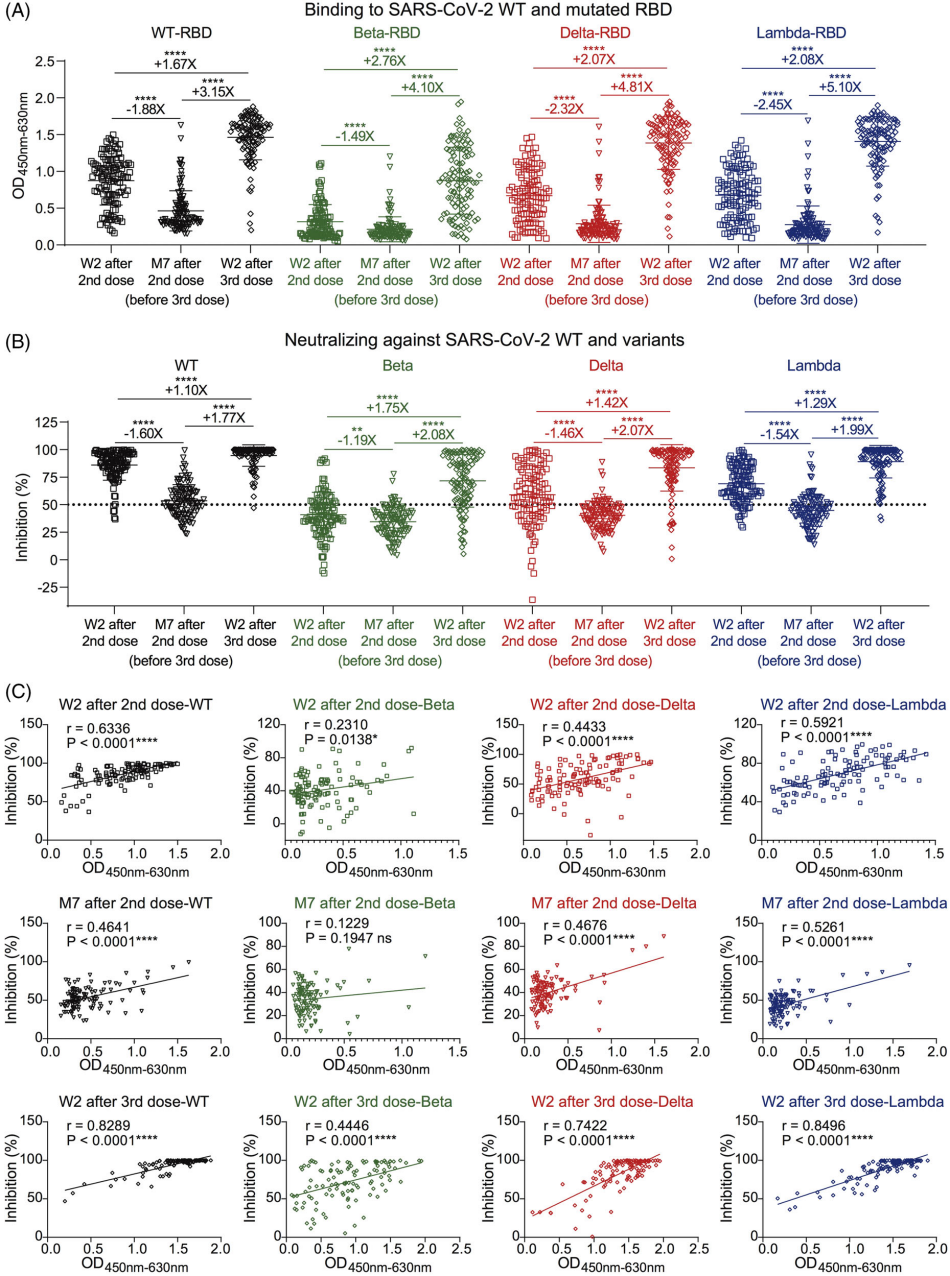
Potent binding and neutralizing antibodies against SARS‐CoV‐2 variants induced by a third dose of inactivated vaccine. (A) ELISA binding of 113 donors at three follow‐up visits to SARS‐CoV‐2 WT and mutated RBD proteins. Plasma samples were tested at a dilution of 1:20. ELISA, enzyme‐linked immunosorbent assay; O.D., optical density. (B) Neutralizing activities of 113 donors at three follow‐up visits against SARS‐CoV‐2 WT, beta, delta, and lambda variants. The inhibition of 50% is indicated by a horizontal dashed line. (C) Correlation analysis between binding and neutralizing activities against SARS‐CoV‐2 WT and variants of 113 donors at three follow‐up visits. The correlation was analysed using GraphPad Prism 8.0 software by linear regression model. ‘+’ indicates increased binding or neutralizing activity, ‘–’ indicates decreased binding or neutralizing activity. ‘X’ indicates fold change. *p < .05; **p < .01; ****p < .0001; ns, not significant

The memory B cell (MBC) response is another important type of immune protection, whose quantity and quality contribute to the speed and potency of the immune system responding to viral reinfection.^9^ Therefore, we randomly selected 24 individuals before the third vaccination (at week 2 and month 7 after the second vaccination) and week 2 after the third vaccination to detect SARS‐CoV‐2 RBD‐specific MBCs (Figure S4). Although plasma anti‐RBD IgG was gradually decreased over time, the percentage of RBD‐specific MBCs maintained a similar level at month 7 as that at week 2 after the second vaccination. Notably, the percentage rapidly increased after the third vaccination, which was significantly higher than that at week 2 after the second vaccination and before the third vaccination. The mean fluorescence intensity (MFI) of RBD‐binding MBCs was also significantly enhanced by the third vaccination (4799 vs. 2951 and 2680 in APC, 8894 vs. 4516 and 4352 in PE) (Figure 3A). These data indicated that the third vaccination not only increased the proportion of MBCs but also enhanced the affinity of MBCs to RBD.

**FIGURE 3:**
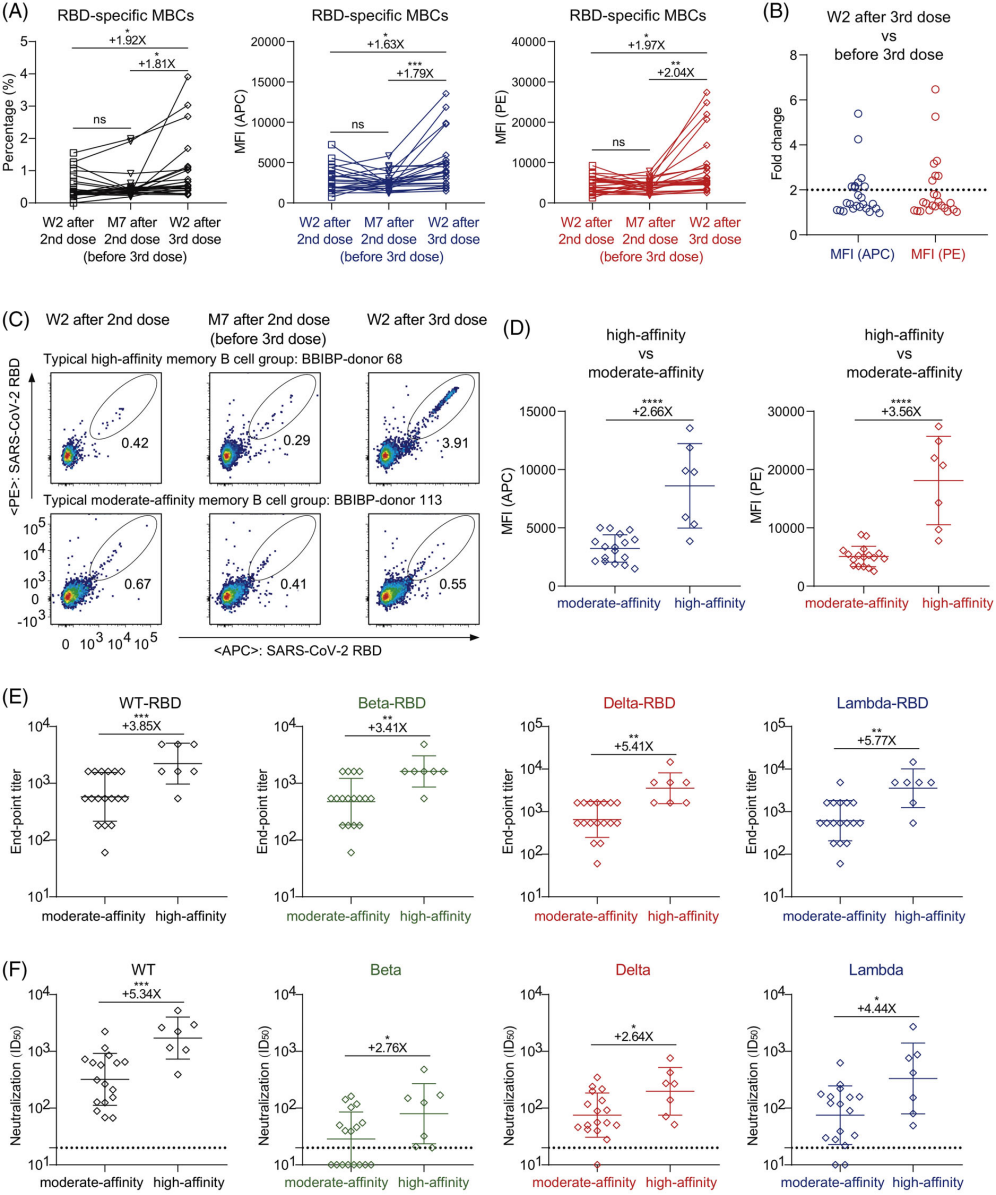
High‐affinity RBD‐specific memory B cells elicited by a third dose of inactivated vaccine. (A) The percentage (left), mean fluorescence intensity (MFI) of APC (middle), and MFI of PE (right) of RBD‐specific MBCs (CD19^+^CD3^−^CD8^−^CD14^−^CD27^+^IgG^+^SARS‐CoV‐2‐RBD^+^ cells) of randomly selected 24 donors with three follow‐up visits. (B) The fold change of MFI in both APC and PE of RBD‐specific MBCs between week 2 after third vaccination and before third vaccination. A cut‐off of twofold is indicated by the horizontal dashed line. High‐affinity group: fold change >2, moderate‐affinity group: fold change <2. (C) The typical display of high‐affinity and moderate‐affinity RBD‐specific MBCs of two donors with three follow‐up visits (high: BBIBP‐donor 68, moderate: BBIBP‐donor 113). (D) Comparison of MFI in both APC (left) and PE (right) of RBD‐specific MBCs at week 2 after third vaccination between the high‐affinity group (*n* = 7) and the moderate‐affinity group (*n* = 17). (E) The end‐point titres of binding IgG to SARS‐CoV‐2 WT, beta, delta, and lambda RBD proteins at week 2 after third vaccination in the high‐affinity and moderate‐affinity groups. (F) The geometric mean titres of nAbs against SARS‐CoV‐2 WT, beta, delta, and lambda pseudoviruses at week 2 after third vaccination in the high‐affinity and moderate‐affinity groups. A cut‐off of 1:20 dilution is indicated by a horizontal dashed line. ID_50_, 50% inhibitory dilution. ‘+’ indicates an increase. ‘X’ indicates fold change. *p < .05; **p < .01; ***p < .001; ****p < .0001; ns, not significant

More importantly, we found that the third dose of vaccine‐induced extremely high‐affinity MBCs in seven individuals, whose MFIs of APC and PE on RBD‐binding MBCs were both more than two‐fold higher than before the third vaccination (Figure 3B–D). After the third vaccination, the geometric mean end‐point titres of the plasma IgG were significantly higher in the high‐affinity group than those in the moderate‐affinity group against WT and mutated RBD proteins (Figure 3E; Figure S5). Similarly, the neutralizing activities of plasma were significantly higher in the high‐affinity group (Figure 3F; Figure S6). Therefore, the third vaccination indeed elicited more potent nAbs and high‐affinity MBCs, suggesting a persistent antibody immunity to SARS‐CoV‐2 variants.

In conclusion, our data highlighted the challenges for vaccine recipients who have received complete immunization for more than 6 months. They are at risk of breakthrough infection by SARS‐CoV‐2 variants. Based on the cohort of volunteers with a long follow‐up time, we emphasized the importance of the third dose of inactivated vaccine to confer higher neutralization against emerging variants. Long‐term follow‐up will evaluate the duration of the antibody response elicited by the third vaccination in future studies.

## CONFLICT OF INTEREST

The authors declare no conflict of interest.

## Supporting information

Supporting Information
