# Supplementary material for: Potent antibody immunity to SARS‐CoV‐2 variants elicited by a third dose of inactivated vaccine
Source: Clin Transl Med. 2022 Feb 27;12(2):e732. doi: 10.1002/ctm2.732 (PMC8882237; doi:10.1002/ctm2.732)
Supplement: Supplementary file 1 — Supporting Information [file CTM2-12-e732-s001.docx]

**Supplementary information**

**Materials and Methods**

**Study approval and blood samples**

This study was approved by the Ethics Committee of Shenzhen Third People’s Hospital, China (approval number: 2020-030). All participants who received vaccines and donated blood samples voluntarily had provided written informed consent for sample collection and subsequent analysis. All plasma and peripheral blood mononuclear cells (PBMCs) from individuals who received two or three doses of inactivated SARS-CoV-2 vaccines (BBIBP-CorV, the Sinopharm COVID-19 vaccine, Beijing Institute of Biological Products Co., Ltd) were collected at different time points of follow-up from the Biobank of the Shenzhen Third People’s Hospital. The blood samples of healthy donors were collected from non-vaccinated donors before the COVID-19 pandemic in 2019. All plasma samples were stored at -80 °C and heat-inactivated at 56 °C for 1 h before use. The SARS-CoV-2 RBD-specific plasma IgG and IgM were measured using the Chemiluminescence immunoassay kit (Beijing Wantai Biotech)^1^, which were displayed in cut-off-index (COI)^2^. PBMCs were maintained in freezing medium and stored in liquid nitrogen.

**Enzyme linked immunosorbent assay (ELISA)**

SARS-CoV-2 wild-type (WT) and mutated (Beta: K417N-E484K-N501Y, Delta: L452R-T478K, Lambda: L452Q-F490S) RBD proteins (Sino Biological) were separately coated into 96-well plates at 4 °C overnight. The plates were washed with PBST buffer and blocked with 5% skim milk and 2% bovine albumin in PBS at room temperature (RT) for 1 h. Plasma samples were diluted at 1:20, added to the wells, and then incubated at 37 °C for 1 h. The plates were washed, and HRP-conjugated goat anti-human IgG antibodies (ZSGB-BIO) were added and then incubated at 37 °C for 30 mins. Finally, the TMB substrate (Sangon Biotech) was added to the wells and incubated at RT for 5 mins, and the reaction was stopped with 2 M H_2_SO_4_. The readout was detected at wavelengths of 450 nm and 630 nm. For titration of the end-point titers of binding antibodies, plasma samples were serially diluted 3-fold from 1:20 to 1:43740 and then added to the plates. The following steps were the same as those mentioned above. A cutoff was set as an OD_450nm-630nm_ value of 0.100. The end-point titer was defined as the last dilution whose OD_450nm-630nm_ value was over 0.100.

**SARS-CoV-2** **pseudovirus-based neutralizing assay**

SARS-CoV-2 pseudovirus was generated by cotransfection of HEK-293T cells with SARS-CoV-2 spike-expressing plasmid and an env-deficient HIV-1 backbone vector (pNL4-3.Luc.R-E-). Two days post transfection, the culture supernatant was harvested, clarified by centrifugation, filtered and stored at -80 °C. To determine the neutralizing activity, plasma samples were serially diluted and incubated with an equal volume of SARS-CoV-2 pseudovirus at 37 °C for 1 h. HEK-293T-hACE2 cells were subsequently added to the plates. After a 48 h incubation, the culture medium was removed, and 100 μL of Bright-Lite Luciferase reagent (Vazyme Biotech) was added to the cells. After a 2 min incubation at RT, 90 μl of cell lysate was transferred to 96-well white solid plates for measurements of luminescence using the Varioskan™ LUX multimode microplate reader (Thermo Fisher Scientific). The 50% inhibitory dilution (ID_50_) was calculated using GraphPad Prism 8.0 software by log (inhibitor) vs. normalized response - Variable slope (four parameters) model.

Detailed sequence information of spike proteins used in this study were listed below, respectively.

SARS-CoV-2 wild-type (WT):

Wuhan-Hu-1, accession number: NC_045512;

SARS-CoV-2 Beta:

D80A, D251G, 242-243del, K417N, E484K, N501Y, D614G, A701V;

SARS-CoV-2 Delta:

T19R, G142D, 157-158del, L452R, T478K, D614G, P681R, D950N;

SARS-CoV-2 Lambda:

G75V, T76I, R246N, 247-253del, L452Q, F490S, D614G, and T859N.

**Flow cytometric analysis of RBD-specific memory B cells**

Thawed PBMCs were stained with an antibody cocktail consisting of CD19-PE-Cy7, CD3-Pacific Blue, CD8-Pacific Blue, CD14-Pacific Blue, CD27-APC-H7, and IgG-FITC (all from BD Biosciences) to gate IgG^+^ memory B cells. SARS-CoV-2 WT RBD with His tag (Sino Biological) was used as a probe to target antigen-specific B cells. Two anti-His secondary antibodies separately labeled with APC and PE (Abcam) were both used to recognize the RBD bait and exclude nonspecific staining. A LIVE/DEAD Fixable Dead Cell Stain Kit (Invitrogen) was used to exclude dead cells. Flow cytometric data were acquired on an Aria II flow cytometer (BD Biosciences) and analyzed using FlowJo software (TreeStar).

**Statistical analysis**

Statistical analysis was performed with paired or unpaired *t* tests (Two-tailed) using GraphPad Prism 8.0 software. *, P < 0.05; **, P < 0.01; ***, P < 0.001; ****, P < 0.0001; ns, not significant.

**References**

1 Zhao, J. *et al.* Antibody Responses to SARS-CoV-2 in Patients With Novel Coronavirus Disease 2019. *Clin Infect Dis* **71**, 2027-2034, doi:10.1093/cid/ciaa344 (2020).

2 Yu, S. *et al.* Distinct kinetics of immunoglobulin isotypes reveal early diagnosis and disease severity of COVID-19: A 6-month follow-up. *Clin Transl Med* **11**, e342, doi:10.1002/ctm2.342 (2021).


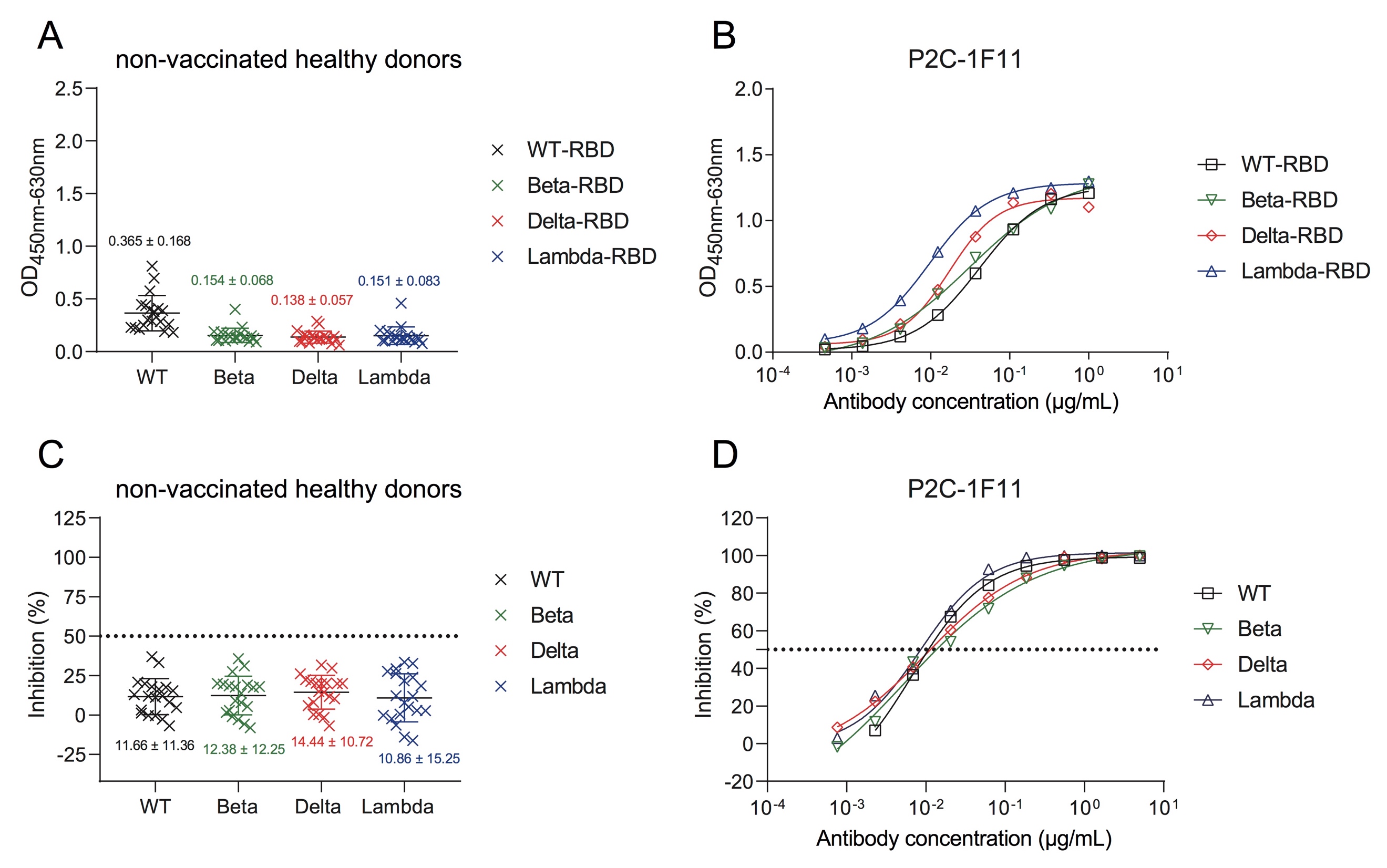


**Figure S1. ELISA and neutralization profiles of non-vaccinated healthy donor plasma and a positive control mAb.**

ELISA binding of 20 non-vaccinated healthy donor plasma samples collected prior to COVID-19 pandemic **(A)** and a positive control mAb **(B)** to SARS-CoV-2 WT, Beta, Delta, and Lambda RBD proteins. Neutralizing activities of 20 non-vaccinated healthy donor plasma samples collected prior to COVID-19 pandemic **(C)** and a positive control mAb **(D)** against SARS-CoV-2 pseudoviruses of WT, Beta, Delta, and Lambda variants. Healthy donor plasma samples were tested at a dilution of 1:20. A positive control mAb (P2C-1F11) was serially 3-fold diluted from 1 μg/mL in ELISA and 5 μg/mL in neutralizing assay. All experiments were performed in duplicate and the mean ± SD values were shown.


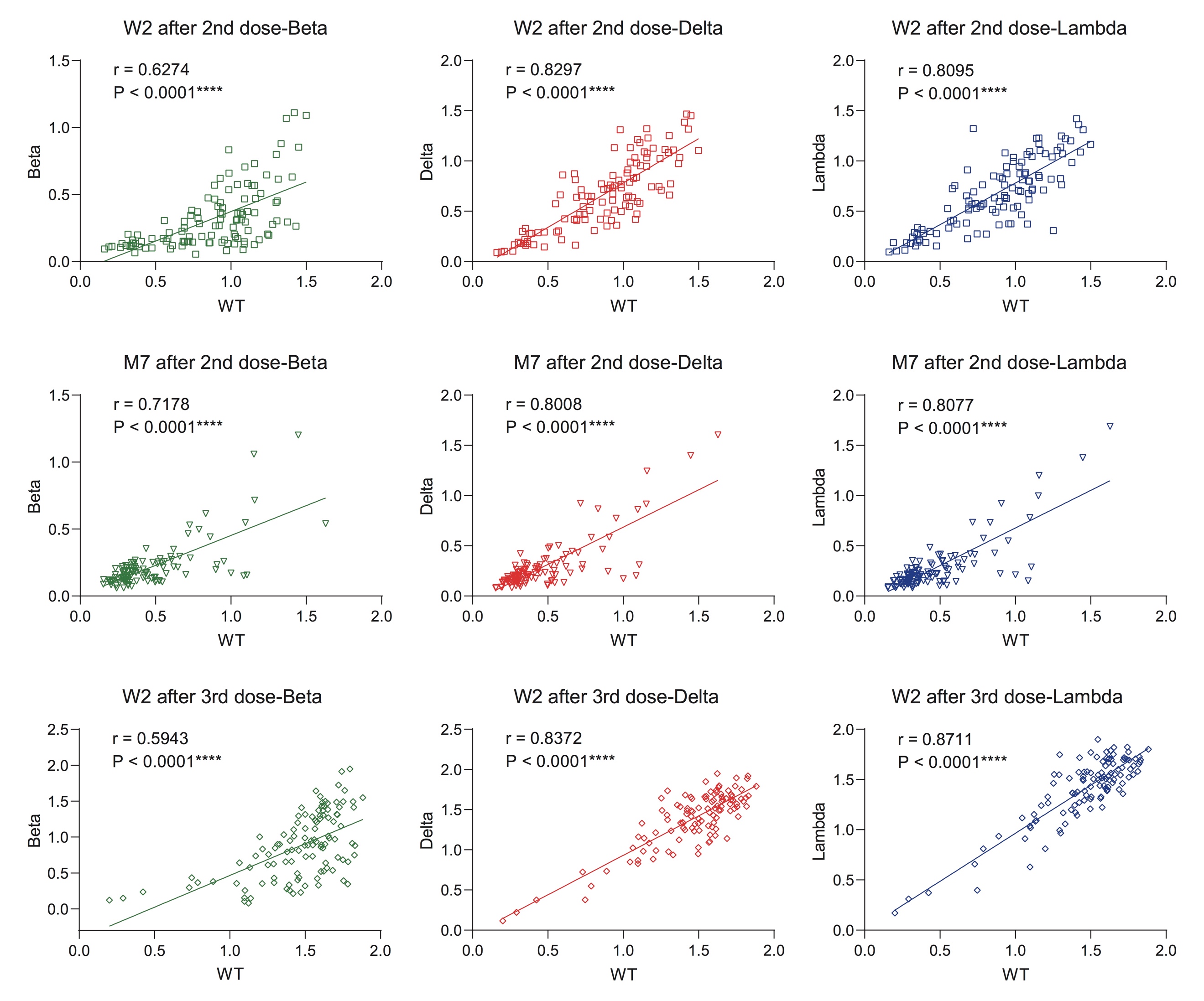


**Figure S2. Correlation analysis between binding activities to** **SARS-CoV-2 WT and mutated** **(Beta, Delta, and Lambda) RBD proteins of 113** **identical participants at three follow-up visits.**

The correlation was analyzed using GraphPad Prism 8.0 software by Linear Regression model.


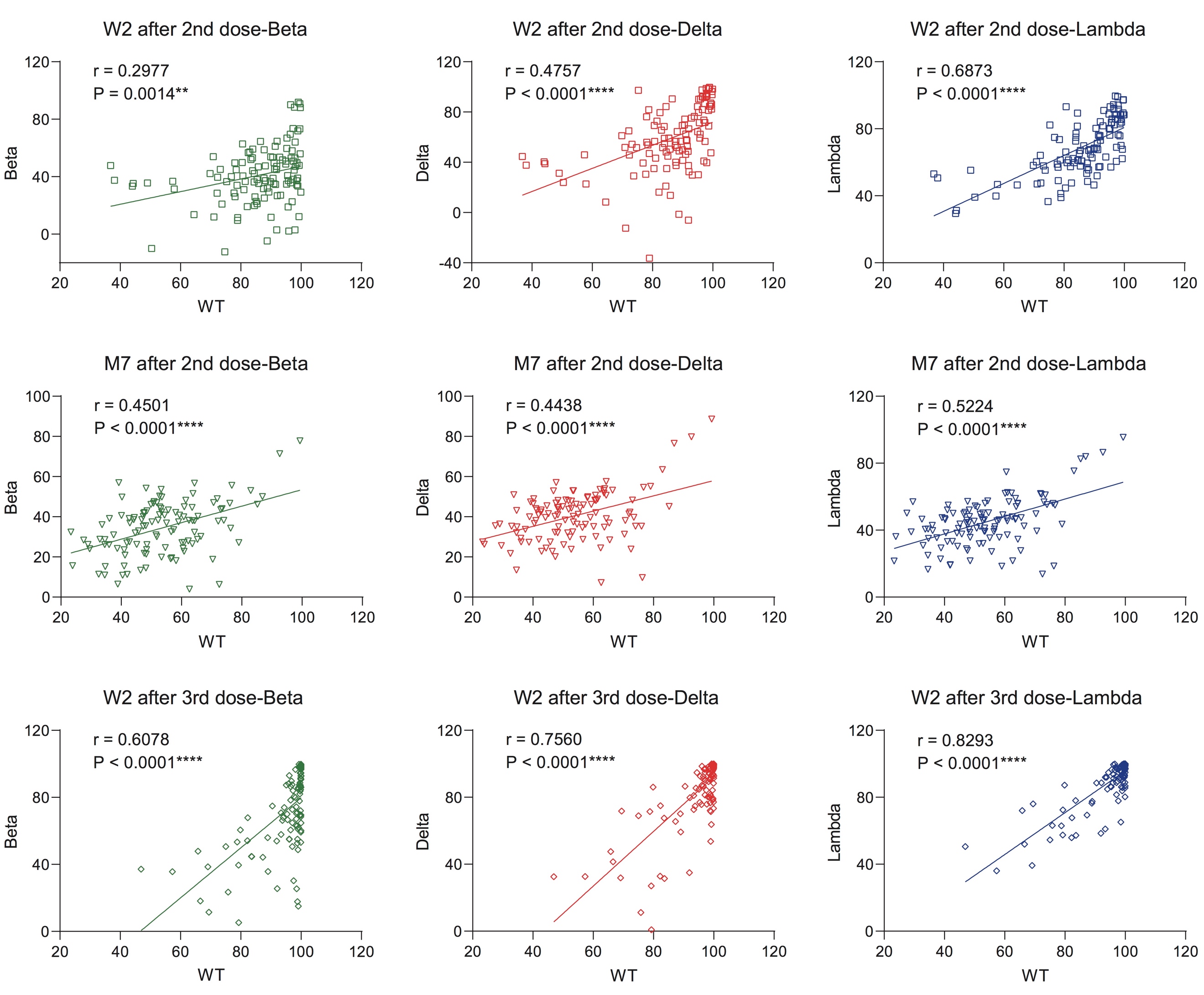


**Figure S3. Correlation analysis between neutralizing activities against SARS-CoV-2 WT and variants (Beta, Delta, and Lambda) of 113 identical participants at three follow-up visits.**

The correlation was analyzed using GraphPad Prism 8.0 software by Linear Regression model.


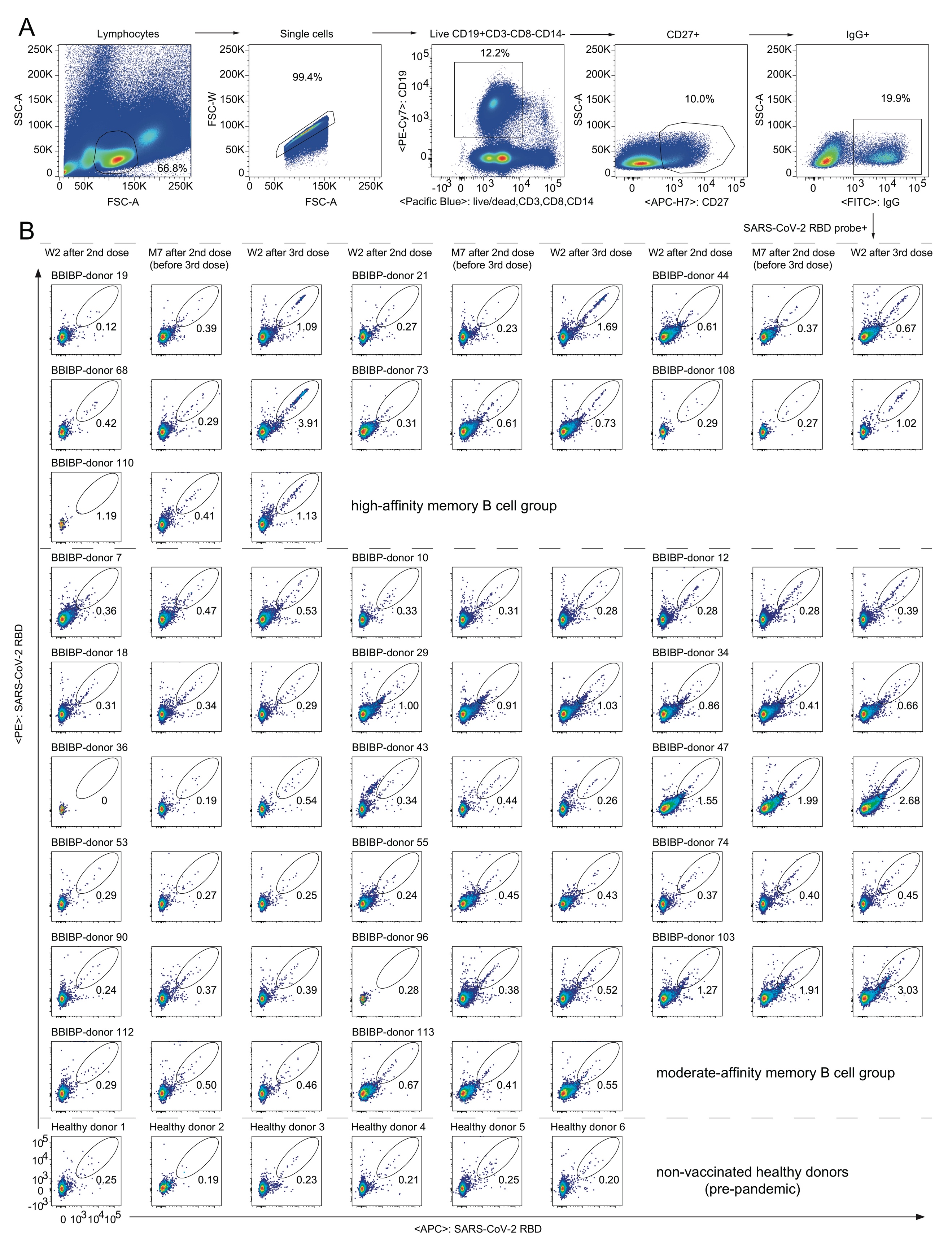


**Figure S4. The gating strategy for identification of SARS-CoV-2 WT RBD-specific memory B cells by FACS.**

**(A)** Single B cells were gated as CD19^+^CD3^-^CD8^-^CD14^-^CD27^+^IgG^+^. **(B)** Flow cytometry showing the percentage of double-positive (APC^+^PE^+^) RBD-binding memory B cells of randomly selected 24 identical participants at three follow-up visits and 6 non-vaccinated healthy donors.


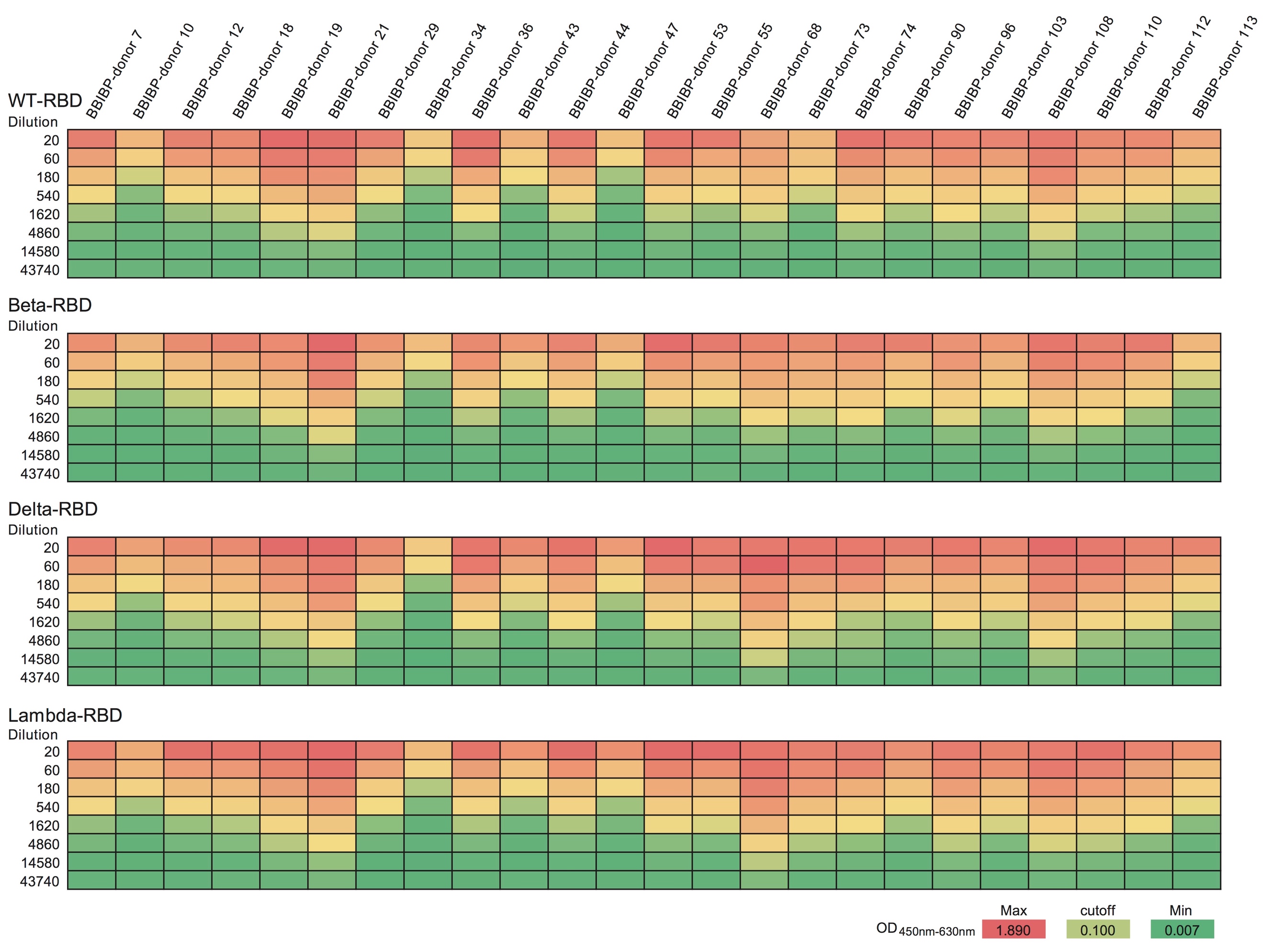


**Figure S5.** **ELISA binding of 24 vaccinee plasma samples at Week 2 after third vaccination to SARS-CoV-2 WT and mutated (Beta, Delta, and Lambda) RBD proteins.**

All plasma samples were serially 3-fold diluted from 1:20. The assay was performed in duplicate and the mean value in each dilution was shown. The cut-off value was set as an OD_450nm-630nm_ value of 0.100 and the end-point titer was defined as the last dilution whose OD_450nm-630nm_ value was more than 0.100.


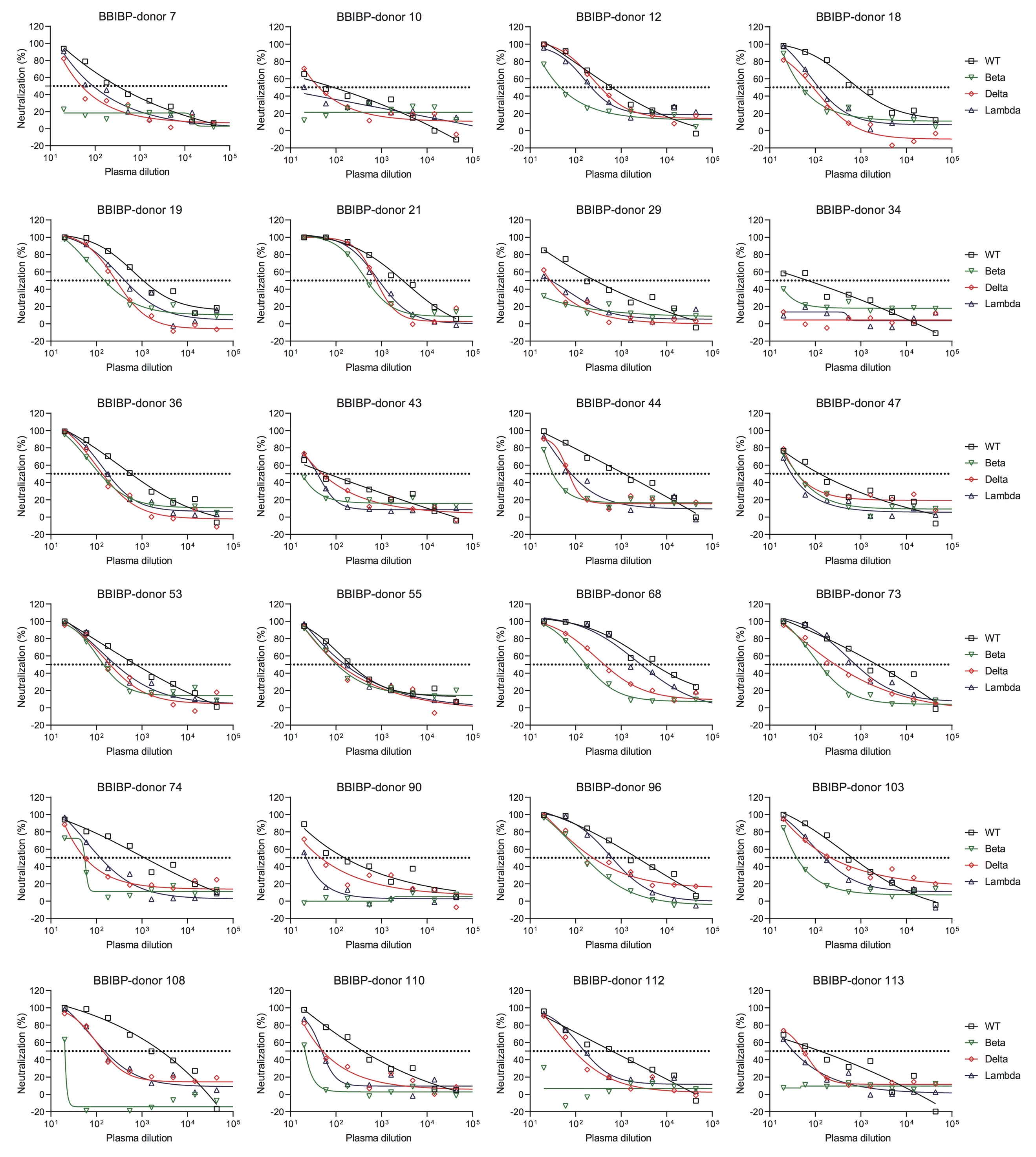


**Figure S6. Neutralization curves of 24 vaccinee plasma samples at Week 2 after third vaccination against SARS-CoV-2 pseudoviruses of WT and variants (Beta, Delta, and Lambda).**

All plasma samples were serially 3-fold diluted from 1:20. The assay was performed in duplicate and the mean inhibition in each dilution was shown. A 50% reduction in viral infectivity was indicated by a horizontal dashed line.
